# Supplementary material for: Medium-Chain Fatty Acids Extracted from Black Soldier Fly (Hermetia illucens) Larvae Prevents High-Fat Diet-Induced Obesity In Vivo in C57BL/6J Mice
Source: Animals (Basel). 2025 May 10;15(10):1384. doi: 10.3390/ani15101384 (PMC12108495; doi:10.3390/ani15101384)
Supplement: Supplementary file 1 [file animals-15-01384-s001.zip › animals-3591059-supplementary.pdf]

Supplementary. Table S1. Proximate macronutrient composition of diets

Diet	Energy (kcal/g)	Protein (% kcal)	Fat (% kcal)	Carbohydrate (% kal)
ND	3.85	20%	10%	70%
HFD	5.24	20%	60%	20%
HFD_MCFA	5.24	20%	60%	20%
